# The impact of the European Association of Endoscopic Surgery research grant scheme—a mixed qualitative quantitative methodology study protocol

**DOI:** 10.3389/fsurg.2023.1197103

**Published:** 2023-06-19

**Authors:** Adam McClean, Bright Huo, Jing Yi Kwan, Judith Long, Josephine Walshaw, Mina Mesri, Nader Francis, Tan H. Arulampalam, Ian Chetter, Marina Yiasemidou

**Affiliations:** ^1^ Clinical Fellow General Surgery, Bradford Teaching Hospitals, Bradford, United Kingdom; ^2^ Faculty of Medicine, Dalhousie University, Halifax, NS, Canada; ^3^ NIHR Academic Clinical Fellow, Vascular Surgery, Leeds Teaching Hospitals, Leeds, United Kingdom; ^4^ Research Manager, Academic Vascular Surgical Unit, Hull University Teaching Hospitals NHS Trust, Hull, United Kingdom; ^5^ Department of Health Research, University of York, York, United kingdom; ^6^ Department of Medicine, Hull York Medical School, York, United kingdom; ^7^ Consultant Colorectal Surgeon, Department of Surgery, Yeovil District Hospital NHS Foundation Trust, Honorary Professor of Surgery UCL, Yeovil, United Kingdom; ^8^ Consultant Colorectal Surgeon, ICENI Centre, Colchester Hospital University NHS Foundation Trust, Colchester, Essex, United Kingdom; ^9^ Professor of Surgery, Hull University Teaching Hospital, Hull, United Kingdom; ^10^ Department of Medicine, NIHR Academic Clinical Lecturer in General Surgery, Hull York Medical School, Hull, United Kingdom

**Keywords:** reseach, reseach initiative, funding, laparoscopic surgery, educational research, basic research, clinical research, trials

## Abstract

**Background:**

The European Association of Endoscopic Surgery (EAES) is a surgical society who promotes the development and expansion of minimally invasive surgery to surgeons and surgical trainees. It does so through its activities in education, training, and research. The EAES research committee aims to promote the highest quality clinical research in endoscopic and minimally invasive surgery. They have provided grant funding since 2009 in education, surgery, and basic science. Despite the success and longevity of the scheme, the academic and non-academic impact of the research funding scheme has not been evaluated.

**Aims:**

The primary aim of this project is to assess the short, long term academic and real world impact of the EAES funding scheme. The secondary aims are to identify barriers and facilitators for achieving good impact.

**Methods:**

This will be a mixed qualitative and quantitative study. Semi-structured interviews will be performed with previous grant recipients. The questions for the interviews will be selected after a consensus is achieved amongst the members of the steering committee of this project. The responses will be transcribed and thematic analysis will be applied. The results of the thematic analysis will be used to populate a questionnaire which will be disseminated to grant recipients. This study is kindly funded by the EAES.

**Discussion:**

The first question this project is expected to answer is whether the EAES research funding scheme had a significant positive impact on research output, career progression but also non-academic output such as change in clinical guidelines, healthcare quality and cost-effectiveness improvement. This project however is also expected to identify facilitators and barriers to successful completion of projects and to achieving high impact. This will inform EAES and the rest of the surgical and academic communities as to how clinicians would like to be supported when conducting research. There should also be a positive and decisive change towards removing factors that hinder the timely and successful completion of projects.

## 1. Introduction

The European Association of Endoscopic Surgery (EAES) is a surgical society who promotes the development and expansion of minimally invasive surgery to surgeons and surgical trainees (1). It does so through its activities in education, training, and research. The EAES research committee aims to promote the highest quality clinical research in endoscopic and minimally invasive surgery. They have provided grant funding since 2009 in education, surgery, and basic science (2). Despite the success and longevity of the scheme, the academic and non-academic impact of the research funding scheme has not been evaluated.

The importance of establishing the impact of a research funding scheme is multifaceted as clinicians, policymakers, and researchers benefit from strategic investment to promote high-quality research (3). Research funding has been found to contribute directly to successful academic publications and conference presentations, and facilitate progression of successful applicants into academic career paths (4, 5). Correspondingly, research stakeholders and independent charities have introduced funding policies to promote what is frequently referred to as scientific excellence (6). Providing competitive funding based on the potential to produce high quality academic output is a popular method of funds allocation (7), but this may to some extent ignore the societal relevance and the need for research to produce positive change beyond the constraints of academia (6).

The established research funding process based on the notion of “excellence”, is used widely, at least in the western world (6); however, it has occasionally been under scrutiny. Authors argue that the concept of excellence is dependent upon whomever defines it, as a validated definition of this within academia does not exist (8, 9). Others believe that it promotes a culture of competition and an outcome focused ethos, often at odds with impactful research outputs (10). Moreover, there have been reports that up to 85% of clinical, health services, and basic science research is avoidably wasted (11).

The Economic and Social Research Council (ESRC) defines research impact as “the demonstrable contribution that excellent research makes to society and the economy” (12). The impact of research can be divided into: Instrumental impact, i.e., influencing policy, practices or services, conceptual impact; understanding policy matters and reframing debate and building capability through technical or personal skill development (12).

Although several models have been proposed for assessing research impact (13), a review by Raftery et al. (14) identified the “payback model” by Buxton and Hanney (15) as the dominant one. Impact according to this model focuses in four areas:

(i) Knowledge production (e.g., publications, presentations),
(ii) Research capacity building (e.g., training new academics)
(iii) Informing policy and product development (e.g., influencing policies and guidelines)
(iv) Health system benefits (e.g., cost savings), economic benefits (e.g., commercial spin-outs) (15).

This model combines areas of academic excellence such as presentations and publications, as well as impact outside academia (e.g., informing policy and economic benefits) (15).

Though identifying the impact of research is important, it is equally vital to identify factors promoting or impeding high-quality research (3). A Lancet series recommends that efforts should be made to identify factors associated with successful research initiatives, recognising the importance of ensuring that research investments yield productive initiatives (11). Farrokhyar et al. (3) showed that assessing the scientific productivity of organisational grant funding can identify the characteristics of grant awardees, as well as the influence of funding on career growth (3).

The International School on Research Impact Assessment (ISRIA) was established by research organisations recognising the need for research impact strategies and uniform assessment efforts based on the robust and reproducible evaluation methods (16). ISRIA developed a series of best practice guidelines (17), encouraging organisations to (1) analyse the research context, (2) reflect on the purpose of RIA, (3) identify stakeholders' needs, (4) engage with the research community, (5) choose appropriate conceptual frameworks, (6) choose appropriate evaluation methods and data sources, (7) choose indicators and metrics responsibly, (8) consider ethics and conflicts of interest, (9) communicate results, and (10) share learning (17).

In line with the ISRIA guidelines the EAES research committee is embarking on this project aiming to evaluate the academic and non-academic impact of their research funding scheme and to identify barriers and facilitators for successful completion of research projects and for achieving the good impact.

For this project that is kindly funded by EAES; The aims and objectives of the project are as follows:

• Primary Aim: Assess the short, long and real world impact of the EAES funding scheme
  ○ Objective 1: Assess the academic impact of the EAES research funding scheme
  ○ Objective 2: Assess the non-academic impact of the EAES research funding scheme
• Secondary Aim: Identify barriers and facilitators for achieving good impact.
  ○ Establish the completion rate of projects funded
  ○ Identify facilitators for achieving high impact
  ○ Identify barriers for the completion of research projects and hence achieving high impact

## 2. Methods and analysis

Research impact can be established with in-depth analysis of the areas described above which can be roughly categorised to academic impact and impact outside of academia. Such an attempt would involve both qualitative and quantitative methods. This would include semi-structured interviews with researchers (18), questionnaires and assessing objective, quantified measures of impact (e.g., number of publications). In addition to the above, through mixed methodology, further impact areas can be identified.

This will be a mixed qualitative, quantitative study, as it will encompass both semi-structured interviews and a questionnaire.

The study will be performed in several stages.

### 2.1. Stage 1: preparing the questionnaire

The survey will be designed with the Checklist for Reporting Results of Internet E-Surveys (CHERRIES) developed by the Enhancing the QUAlity and Transparency Of health Research Network (Equator) (19) in mind. This consists of eight topics:

1. Design: The target population will be previous recipients of the EAES research grants. They will be invited directly via email.
2. Institutional approval, informed consent and data protection: This project has been approved by Hull and York Medical School Ethics Committee. Participants will be kindly requested to sign an informed consent. It will be made clear that participation is voluntary and that they can withdraw from the project whenever they wish, without having to provide a reason. However, any data they may have provided up to the point of withdrawal may be used in the final analysis. No identifiable data will be stored and the responses to the survey will be anonymous.
3. Development of questionnaire. Based on the four areas proposed above, the steering committee of this project is expected to reach a consensus on questionnaire questions. The process will involve each member of the steering committee independently reviewing a pre-drafted list of questions. Supplementary Appendix 1 demonstrates the list of draft questions for the committee's review. The committee will then have a series of virtual meetings, until an absolute (100%) consensus is reached. The number of questions is not rigidly defined, therefore, if the members of the steering group feel that questions need to be added or removed, this can be done after the necessary consensus is reached.
4. Recruitment process and description of the sample having access to the questionnaire. The participants will be recruited after email invitation through the EAES mailing list.
5. Survey administration. The study will be created and disseminated through Google® docs. The link of the survey will be shared with investigators via email. As mentioned above, participation will be voluntary. Participants will be given an incentive to complete the survey, as one of the responders will be randomly selected for a travel grant to the next EAES conference. The survey will remain open for 4 weeks. The number of items will be determined at the designing process however, the entirety of the questionnaire will be in one page only. This will allow participants the ability to go back and change their answers, they can also scroll back and review the final version of their answers before they submit the completed questionnaire.
6. Response rate. This will be easily calculated as the proposed participant number will be known at the beginning of the survey.
7. Preventing multiple entries from the same individual. Each responder will have to provide details about their project therefore, duplication is unlikely. However, IP identification will be used to ensure there is no duplication no two entries from the same IPs will be allowed.
8. Analysis. Incomplete questionnaires are not expected as all questions requiring an answer are made compulsory, therefore the user cannot progress to the next question unless the previous one was answered. The answers will be extracted in an Excel (Microsoft®) sheet, analysed and presented with appropriate descriptive statistics.

### 2.2. Stage 2: delivering the questionnaire

Grant recipients of the past years will be invited to answer the questionnaire. This will be done through the emailing list of EAES. The participants will be asked whether they would like to volunteer to participate in phase 2 which will be semi-structured interviews aiming to further define questionnaire answers.

### 2.3. Stage 3: preparation and delivery of semi-structured interviews

The steering committee of the project will be asked to review the raw data from the survey results and suggest 5 questions which they feel will further define the results of the survey. Once consensus is established, semi-structured interviews will be conducted with 6–10 randomly selected survey responders stemming from the novel method of information power for sample sizing in qualitative research methodology by Malterud and colleagues (20). The interviews will be conducted by researchers with previous relevant experience.

The transcripts of the interviews will be transcribed verbatim and analysed by two independent assessors (Marina Yiasemidou, Judith Long) who have previous experience with such analysis.

Target population:

Past EAES Grant recipients will be asked to reply to the questionnaire developed by the project's steering committee. Responders will be invited to participate in semi-structured interviews. Participation will be voluntary.

### 2.4. Data collection and analysis

#### 2.4.1. Survey

Data collection will be performed using a bespoke spreadsheet. Besides the answers of the survey, demographics and project characteristics will be collected.

The results of the survey will be reported according to the Checklist for Reporting Results of Internet E-Surveys (CHERRIES) developed by the Enhancing the QUAlity and Transparency Of health Research Network (Equator) (19).

#### 2.4.2. Semi-structured interviews—thematic analysis

The thematic analysis of the semi-structured interviews will be completed in the following steps: (i) familiarisation (ii) coding (iii) generating themes (iv) reviewing themes (v) naming themes (vi) write up of results (18). Familiarisation will occur during the transcribing of the interviews. Coding will involve highlighting sections of the transcript and generating “codes” to describe their content. For example, if the transcript states, “I am not sure” the code can be “uncertainty”. During the next step of generating themes, the codes created will be assessed for patterns amongst them. This process will generate themes; a broader concept than codes. On most occasions, several codes will be combined into a single theme (18). Following this, the themes will be reviewed by the two assessors (MY, JL) to ensure that they are useful and accurate representations of the data.

This may result in themes being divided, combined, discarded or generated. This process will be ultimately validated by the steering committee. The themes developed will then need to be named in a succinct and precise way. The last step of the process will be the write up of the report, which will be prepared and delivered to the steering committee for careful scrutiny (18).

### 2.5. Analysis

#### 2.5.1. Semi-structured interviews—thematic analysis

The themes will be reviewed by the two assessors (MY, JL), a report will be generated and presented to the steering committee. The entire process will be overseen and validated by the steering committee.

#### 2.5.2. Questionnaire

The results of the questionnaire will be presented using descriptive statistics such as frequencies and percentages.

## 3. Discussion

This study will determine whether the EAES research funding scheme had a significant positive impact on research output, career progression but also non-academic output such as change in clinical guidelines, healthcare quality and cost-effectiveness improvement.

This project is also expected to identify facilitators and barriers to successful completion of projects and to achieving high impact. This will inform EAES and the rest of the surgical and academic communities as to how clinicians would like to be supported when conducting research. There should also be a positive and decisive change towards removing factors that hinder the timely and impactful completion of projects. A final report with a list of recommendations to address key issues derived from the questionnaire and semi-structured interviews will be shared with the organizers of the EAES trainee grant funding committee.
