# Supplementary material for: The impact of the European Association of Endoscopic Surgery research grant scheme—a mixed qualitative quantitative methodology study protocol
Source: Front Surg. 2023 Jun 19;10:1197103. doi: 10.3389/fsurg.2023.1197103 (PMC10315820; doi:10.3389/fsurg.2023.1197103)
Supplement: Supplementary file 1 [file Datasheet1.pdf]

Appendix 1. Drafted Objective Questions for Review by the Steering Committee.

1. What is the highest number of citations in a year achieved by a publication derived from your EAES funded project?
2. How many presentations have you achieved through your project?
    a. How many were international presentations?
    b. How many were oral presentations?
    c. How many were invited presentations?
3. Did you achieve any awards related to your project?
4. Were you invited to participate in guidelines committee?
5. Have you been invited to participate into another's team research activities based on your experience gained from the EAES funds research project?
    a. If yes, please describe: [free text]
6. Has your project led to further research?
    a. If yes, please describe: [free text]
        i. Has this led to further funding? If yes, please give details.
7. Has your project generated intellectual Property and/or licencing?
    a. If yes, please provide details: [free text]
8. Was any part of your research cited in guidelines or policy change?
    a. If yes, please provide details: [free text]
9. Has your project generated medical products, a spin-off company, or creative products?
    a. If yes, please provide details: [free text]
10. Since you completed your EAES research study, do you feel that has impacted your training/progress regarding the following?
    a. Career progression (Yes/No)
        i. If yes, please quantify this on the following scale: 1 (not at all) - 7 (great degree)
    b. Confidence in practice
        i. Yes, positive impact
        ii. Yes, negative impact
        iii. No
    c. Further academic career opportunities to engage in clinical or lab-based research
        i. Yes, positive impact
        ii. Yes, negative impact
        iii. No
    d. Academic efficiency
        i. Yes, positive impact
        ii. Yes, negative impact
        iii. No
    e. Clinical progression
        i. Yes, positive impact
        ii. Yes, negative impact
        iii. No
    f. Understanding of research methodology

            i. Yes, positive impact
            ii. Yes, negative impact
            iii. No

11. Do you think that acquiring an EAES research grant helped you with networking?
    a. Yes/No
        i. If yes, please describe: [free text]
        ii. If yes, have you engaged in in collaborative research with investigators you met through this networking?
            1. Yes/No
12. Have you engaged in collaborative research with investigators you met through the above-mentioned networking?
13. Please indicate if you will be willing to have a semi structured interview to help shaping future funding research within the EAES.
14. Please list any additional comments:[free text]
